# A case from the future of HPB surgical oncology: resection of biliary tract cancer after immunotherapy

**DOI:** 10.1093/jscr/rjab414

**Published:** 2021-10-25

**Authors:** Vikas Satyananda, Konstantinos Chouliaras, Leonid Cherkassky, Roderich E Schwarz

**Affiliations:** Department of Surgical Oncology, Roswell Park Comprehensive Cancer Center, Buffalo, NY 14203, USA; Department of Surgical Oncology, Roswell Park Comprehensive Cancer Center, Buffalo, NY 14203, USA; Department of Surgical Oncology, Roswell Park Comprehensive Cancer Center, Buffalo, NY 14203, USA; Department of Surgical Oncology, Roswell Park Comprehensive Cancer Center, Buffalo, NY 14203, USA

## Abstract

Biliary tract cancers (BTCs) have limited response to systemic therapy and poor prognosis. Immunotherapy in BTCs has been investigated in recent years. Here, we report a case of locally advanced, unresectable gallbladder adenocarcinoma that progressed on chemotherapy. The patient was then treated with ipilimumab and nivolumab, which resulted in tumor shrinkage and autoimmune hepatitis, but established technical resectability. He underwent complete resection through extended right hepatectomy with en bloc cholecystectomy bile duct resection, hepatic and portal lymphadenectomy and Roux-Y hepaticojejunostomy reconstruction. The final pathology revealed a pathologic complete response. The scope of operative intervention after immunotherapy is still evolving for BTCs. Establishing resectability in tumors not susceptible to cytotoxic agents but responding to immunotherapy not only facilitates curative intent resection but also enhances the importance of infection prevention through operative stent-free long-term biliary decompression. Immunotherapy may also carry a unique risk profile for post-operative morbidity potential as in this case with autoimmune hepatitis.

## INTRODUCTION

Biliary tract cancers (BTCs), including cancers of gallbladder, extrahepatic and intrahepatic biliary tracts, vary in their etiology, pathophysiology and clinical presentation. Most patients (>65%) present with advanced stage disease, and distant relapse remains common after apparently complete resection [1, 2]. Systemic cytotoxic therapy represents the first line of treatment for unresectable disease, but the response rate is less than 25% [3]. Ongoing trials are exploring novel targeted therapies and immunotherapy, although actionable mutations such as FGFR fusions and IDH mutations are identified in only few BTCs [4].

With continued improvements in systemic therapy, goals, indications and timing of operative intervention in treatment of BCTs are expected to change. We present a case of locally advanced gallbladder carcinoma in which operative options and challenges were determined by the response to immunotherapy.

## CASE REPORT

**Figure 1:**
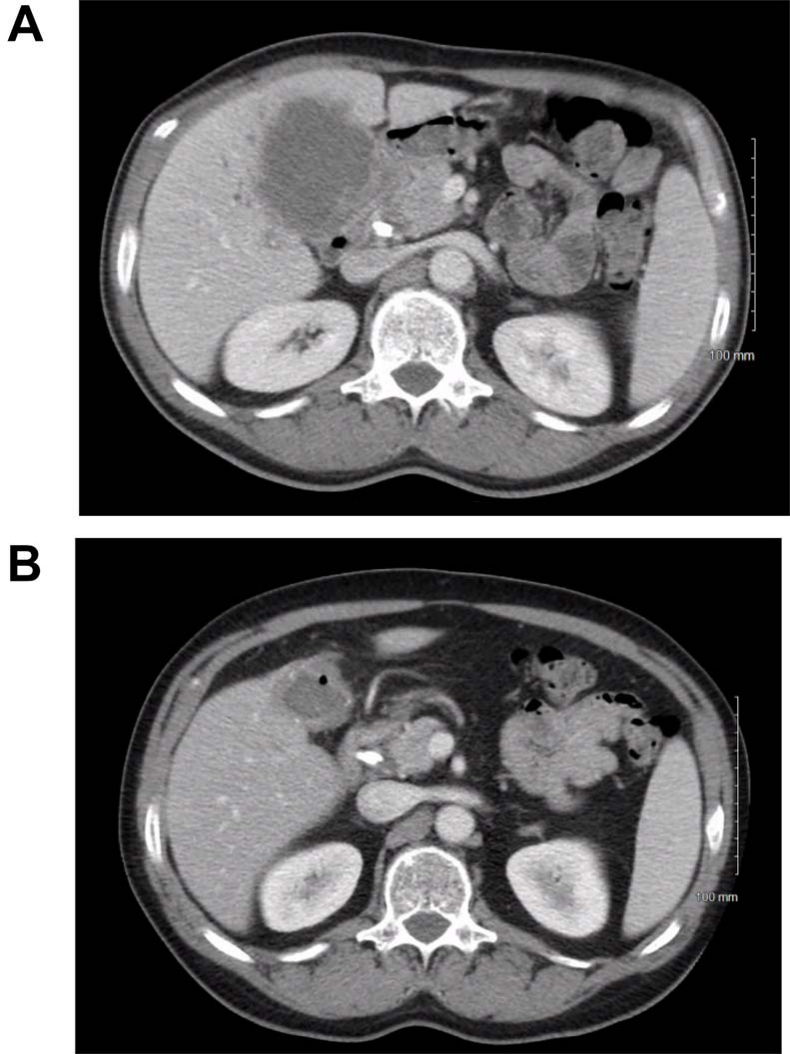
(**A**) CT scan of the abdomen showing the thickened gallbladder as well as a mass involving hilar hepatic parenchyma; (**B**) treatment response after immunotherapy.

A 59-year-old male presented with abdominal pain for several months. Computed tomography (CT) showed a 9.3-cm gallbladder mass with direct extension into the right and left hepatic lobes (Fig. 1A), porta hepatis and mesenteric fat. Biopsy established a poorly differentiated adenocarcinoma of gallbladder origin. Biliary obstruction at the level of the common and cystic duct junction was managed with internal biliary stenting. The process presented as cT3N1M0, locally advanced, and not completely resectable gallbladder adenocarcinoma; serum CA 19–9 level was 2666 U/ml (normal up to 35 U/ml).

**Figure 2:**
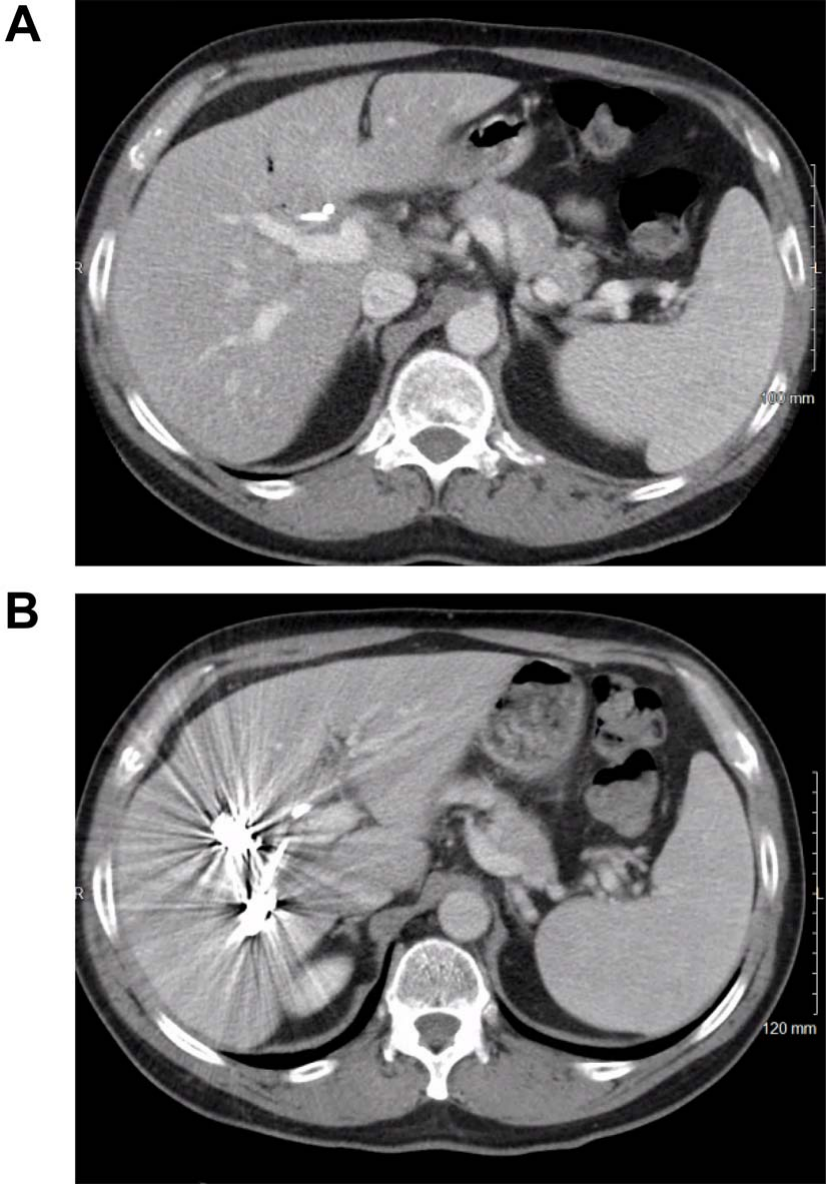
CT scan image of the liver: (**A**) small left lobe prior to portal vein embolization; (**B**) left lobe hypertrophy after right portal vein embolization.

**Figure 3:**
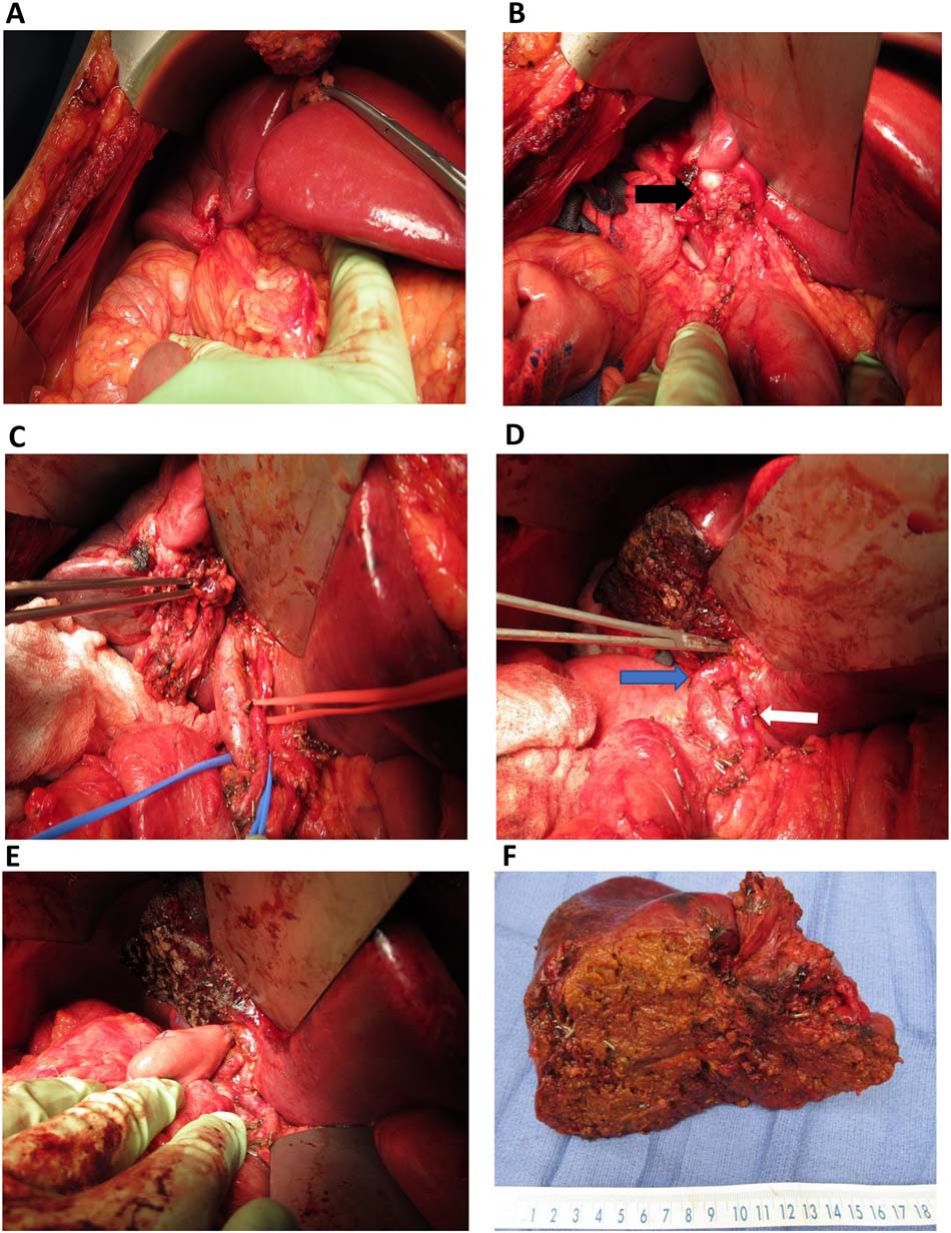
Intra-operative images: (**A**) omentum covering the mass in gallbladder fossa; (**B**) gallbladder mass (black arrow) involving the right-sided hilar structures; (**C**) right-sided portal structures mobilized (in the forceps); (**D**) after resection of the mass, left portal vein (blue arrow) and left hepatic artery (white arrow) are demonstrated. Instrument in left hepatic duct; (**E**) completed hepaticojejunostomy; (F) final specimen.

The patient received systemic chemotherapy with gemcitabine and cisplatin. After more than 2 months, he developed grade 3 neuropathy and experienced several episodes of cholangitis with liver abscess and bacteremia requiring multiple stent exchanges, and systemic therapy was discontinued. He then received trial-based immune check point inhibition with ipilimumab and nivolumab. There was good radiological (Fig. 1B) and tumor marker response (CA 19–9 7.05 U/ml). Immunotherapy had to be stopped after 10 months due to grade 3 autoimmune hepatitis. He was treated with oral steroids and immunosuppression with mycophenolate mofetil, resulting in the normalization of liver function tests. A R0 resection was now deemed a possibility, with a goal of best tumor control and long-term biliary decompression. He underwent right portal vein embolization after which his left lobe future liver remnant grew from 28 to 45% at a kinetic growth rate of 5.6% per week (Fig. 2). He underwent extended right hepatectomy with radical cholecystectomy, portal and hepatic artery lymph node dissection, and Roux-Y hepaticojejunostomy biliary reconstruction (Fig. 3). Pathology examination revealed a 6.5-cm gallbladder and hepatic tissue mass with xanthogranulomatous inflammation and nodular fibrosis, without residual viable tumor, margins free of carcinoma and 0/17 lymph nodes involved, consistent with an ypT0N0M0 gallbladder cancer, R0 resection. After an initial postoperative surgical site infection, he has now fully recovered and is disease-free at 10-month follow-up.

## DISCUSSION

Survival statistics from systemic therapy for gallbladder cancer remain poor; in the ABC-02 trial, doublet therapy of gemcitabine + cisplatin led to a median overall survival of 11.7 months, with radiologic responses only at 26% [3]. Our patient with locally advanced disease had minimal response to this chemotherapy. Immunotherapy has become a novel cornerstone treatment for several solid tumors including those that are deficient in mismatch repair (dMMR), microsatellite instability (MSI) and high mutational burden [5]. While its role in BTCs is not yet clearly established [6], it has been explored in some phase 2 and 3 trials [7]. There are several ongoing trails employing immunotherapy with PD-1 and PD-L1 inhibitors or CTLA4 agents, alone or with cytotoxic therapy, or other targeted molecular therapies [8]. Response rates around 40% have been found in patients with MSI tumors [9]. A complete response of BTCs after pembrolizumab alone has been occasionally reported [10]. However, >97% of BTCs are microsatellite stable, and the durability of clinical responses from checkpoint inhibitors remains unclear. Thus, the indications for such agents remain investigational except in MSI-high/dMMR tumors.

Immunotherapy is also not without complications as observed here. Check point inhibitor-related hepatotoxicity has been reported in up to 16% depending on the class of drugs (CTLA4 > PD/ PD-L1 inhibitors), combination versus monotherapy and dosage [11]. The treatment involves discontinuing the immunotherapy, corticosteroids and immunosuppression [12]. In our patient, hepatotoxicity raised concerns over limited hypertrophy potential and increased risk for postoperative hepatic failure. However, after restoration of normal synthetic liver function, there was no problem with subsequent liver hypertrophy and post-operative regeneration.

The role of resection in patients on immunotherapy is evolving. Aside for curative-intent tumor resection, an important indication rests with resolving biliary tract obstruction and the considerable stent-related infectious morbidity [13, 14]. A meta-analysis found that in patients undergoing pancreatoduodenectomy, pre-operative biliary drainage significantly associated with bacterobilia (86 vs. 25%) and a significantly higher rate of surgical site infections (RR 2.84, *P* < 0.001) [15]. As in our patient with multiple cholangitides requiring inpatient care and stent exchanges, biliary obstruction is a source of long-term morbidity in patients with BTCs for which biliary-enteric anastomosis can be a durable solution. Even in our patient with a complete pathologic response, the operation performed still appears appropriate, given this aspect of controlling long-term biliary infectious morbidity.

This case may serve as an illustration of future role of surgeons in the era of immunotherapy, where indications for operative interventions, resectability and other patient benefits may be all affected differently by the therapeutic response from newer agents. Several challenges still remain. Currently, the only FDA approval for the use of immunotherapy is for dMMR/MSI tumors that constitute less than 2% of all BTCs. Predictors on efficacy and durability of responses to immunotherapy are still lacking. Nevertheless, we expect an emerging role of immunotherapy in a pre-operative setting for locally advanced or borderline resectable BTCs. Operative procedures will quite possibly be selected more often for responding disease, and perhaps at times with a specific indication for biliary tract infection prevention management. Such combined immunotherapy and operative treatment can hopefully establish improved survival and quality of life outcomes for patients with BTCs in the future.

## CONFLICT OF INTEREST STATEMENT

The authors have no conflicts of interest to disclose.

## References

[1] Valle JW . Advances in the treatment of metastatic or unresectable biliary tract cancer. Ann Oncol 2010;21(Suppl 7):vii 345–8. 10.1093/annonc/mdq420 20943640

[2] Schwarz RE, Smith DD. Lymph node dissection impact on staging and survival of extrahepatic cholangiocarcinomas, based on U.S. population data. J Gastrointest Surg 2007;11:158–65. 17390167 10.1007/s11605-006-0018-6

[3] Valle J, Wasan H, Palmer DH, Cunningham D, Anthoney A, Maraveyas A, et al. Cisplatin plus gemcitabine versus gemcitabine for biliary tract cancer. N Engl J Med 2010;362:1273–81. 20375404 10.1056/NEJMoa0908721

[4] Mertens JC, Rizvi S, Gores GJ. Targeting cholangiocarcinoma. Biochim Biophys Acta Mol basis Dis 1864;2018:1454–60. 10.1016/j.bbadis.2017.08.027 PMC6013079 28844952

[5] Le DT, Uram JN, Wang H, Bartlett BR, Kemberling H, Eyring AD, et al. PD-1 blockade in tumors with mismatch-repair deficiency. N Engl J Med 2015;372:2509–20. 26028255 10.1056/NEJMoa1500596 PMC4481136

[6] Rizzo A, Ricci AD, Brandi G. Recent advances of immunotherapy for biliary tract cancer. Expert Rev Gastroenterol Hepatol 2021;15:527–36. 33215952 10.1080/17474124.2021.1853527

[7] Klein O, Kee D, Nagrial A, Markman B, Underhill C, Michael M, et al. Evaluation of combination nivolumab and ipilimumab immunotherapy in patients with advanced biliary tract cancers: subgroup analysis of a phase 2 nonrandomized clinical trial. JAMA Oncol 2020;6:1405–9. 32729929 10.1001/jamaoncol.2020.2814 PMC7393585

[8] Boilève A, Hilmi M, Smolenschi C, Ducreux M, Hollebecque A, Malka D. Immunotherapy in advanced biliary tract cancers. Cancers (Basel) 2021;13:1569–87. 10.3390/cancers13071569 PMC8036747 33805461

[9] Kim RD, Chung V, Alese OB, El-Rayes BF, Li D, Al-Toubah TE, et al. A phase 2 multi-institutional study of nivolumab for patients with advanced refractory biliary tract cancer. JAMA Oncol 2020;6:888–94. 32352498 10.1001/jamaoncol.2020.0930 PMC7193528

[10] Alshari OM, Dawaymeh TA, Tashtush NA, Aleshawi AJ, Al Manasra ARA, Obeidat KA. Completely resolved advanced biliary tract cancer after treatment by pembrolizumab: a report of two cases. Onco Targets Ther 2019;12:5293–8. 31308699 10.2147/OTT.S197559 PMC6615017

[11] Peeraphatdit TB, Wang J, Odenwald MA, Hu S, Hart J, Charlton MR. Hepatotoxicity from immune checkpoint inhibitors: a systematic review and management recommendation. Hepatology 2020;72:315–29. 32167613 10.1002/hep.31227

[12] Cheung V, Gupta T, Payne M, Middleton MR, Collier JD, Simmons A, et al. Immunotherapy-related hepatitis: real-world experience from a tertiary centre. Frontline Gastroenterol 2019;10:364–71. 31656561 10.1136/flgastro-2018-101146 PMC6788136

[13] Sahara K, Merath K, Hyer JM, Paredes AZ, Tsilimigras DI, Mehta R, et al. Impact of preoperative cholangitis on short-term outcomes among patients undergoing liver resection. J Gastrointest Surg 2020;24:2508–16. 31745898 10.1007/s11605-019-04430-7

[14] Schwarz RE . Technical considerations to maintain a low frequency of postoperative biliary stent-associated infections. J Hepato-Biliary-Pancreat Surg 2002;9:93–7. 10.1007/s005340200009 12021902

[15] Müssle B, Hempel S, Kahlert C, Distler M, Weitz J, Welsch T. Prognostic impact of bacterobilia on morbidity and postoperative management after pancreatoduodenectomy: a systematic review and meta-analysis. World J Surg 2018;42:2951–62. 29464345 10.1007/s00268-018-4546-5

